# Agricultural Review

**Published:** 1879-07

**Authors:** 


### AGRICULTURAL REVIEW.

To the scientific character of the nineteenth century we owe the discovery of the conditions which govern vegetable life, and the most important investigations regarding the perpetual fertility of the soil.

Agricultural chemistry was first elevated to a scientific character by the greatest chemist of the age—Professor Justus Von Liebig. In earlier ages many erroneous ideas governed the field of agriculture. We know at the present time that the organic matter of plants is derived from carbonic acid, water, and ammonia, under the reducing influence of the rays of the sun, and that the mineral matter contained in plants—especially as regards the cereals—is not a matter of accident, but on the contrary, they play a very important rôle in the vital process of vegetables.

Carbonic acid and ammonia are, to a certain extent, present in the atmosphere. If this food is provided to a large extent plants will increase in growth and quality, and will reach their greatest development, if the necessary alkalies and phosphates are present in the soil, in a much shorter space of time than they would in the absence of these substances. Both conditions must be united for the welfare of vegetation. But the necessary inorganic salts must be present at the same time. When certain mineral properties are wanting in the soil the nutriment of the plant is imperfect, and the seed will never reach maturity or will not be found at all.

History furnishes us with many examples* where the exhaustion of the soil was followed by the extinction or migration of the people. It is a principle law of nature to return to the soil, under cultivation, that which we take from it in mineral substances, in order to sustain and keep up a perfect state of fertility. Many mineral substances, necessary for the growth of vegetables, are present in great abundance, as, for example, lime, while others, and just the most important one—phosphoric acid—especially necessary for the development of seed, is only contained to a very limited extent in the soil. The excreta of man and animals contain all the mineral matters formerly contained in their food. It is, therefore, obvious and most natural, yet more, an absolute necessity, to return this excreta to the soil.†

In this excreta we have all the means of improving the fertility of the soil, and thereby enhancing our own prosperity; and what an immense amount of this valuable articleis every year distroyed in our cities, and becomes lost to the most important of all purposes—that of agriculture. When we take into consideration that we pay enormous sums annually in transporting from distant climes a guano, which is nothing else than the feces of birds, it does seem most inconsistent, and even ridiculous, that we discard contemptuously the very article in our own cities.

Professor Liebig says: " The coming generation will consider those men as the greatest benefactors of mankind who devote all their efforts to utilize and save the night-soil of the cities." Poudrette works have been established in the United States, Germany, France, and England, but none have ever yet united the sanitary

*Many flourishing countries in olden times, such as Sicily, Sardinia, Palestine, and the formerly fertile coast of Northern Africa, are not far removed from being deserts in our day, and are at the present time inhabited by a demoralized race.

†Fresh feces contain an average of twenty-five per cent. of solid matter and seventy-five per cent of water. The mineral matters consist of one-third of phosphoric acid. Dried feces are, of course, much richer on account of having lost the water. A city of one hundred thousand inhabitants would yield per year one thousand three hundred tons dried feces, containing one hundred and twelve thousand pounds of phosphoric acid.

with the agricultural interests. Some trials have been made to employ iron salts for disinfecting the night-soil, but such a poudrette is almost valueless. Other trials were made with lime, which only caused the loss of the ammonia, and had no disinfecting value whatever. In this regard Dotch's patent process surpasses anything yet introduced. It consists in the application of a prepared earth, containing clay, sulphuric acid, and nitric acid, which is spread in thin layers over the fresh feces.*

By this means not only is the formation of fungoid growth effectually prevented, but also all the ammonia is taken up on account of the sulphuric acid; and the sulphuretted hydrogen developed from the feces will be entirely destroyed by the nitric acid present in the patent earth. There is no better mixture combining both these advantages simultaneously, and, furthermore, the sulphuric acid attacking the clay in the prepared earth will set a part of the silicious matter free, which, assuming a soluble state, is of the greatest direct use to the cereals, inasmuch as it is wanted for the formation of a supporting stem.

Thus we have combined all that is required in a sanitary point of view, with the greatest possible advantage to the purposes of agriculture. We have a poudrette rich in ammonia containing soluble silica without emitting any bad odor. Besides, the nitrogen of the nitric acid, as well as the sulphur of the sulphuric acid, which in the soil, as well as in the poudrette are converted into ammonia salts—are substances directly assimilable by the plants for the formation of albuminous matters, which always contain sulphur and nitrogen. The above-mentioned acids work beneficially upon the soil in rendering mineral matters soluble previously not soluble.

As agriculture is the foundation of human life, and necessary for the development of industry and commerce, as well as science and art, it must be obvious to every reasoning mind that there is no subject of such vital importance, and none which calls more eagerly for the employment of our best efforts to sustain this principal source of our wealth; and this patented process may justly be considered as an important improvement, and worthy of the highest consideration.

---

* The clay and earth are such as to contain no admixture of carbonates, as these would neutralize the acids. $SO_3$, $NO_5$.

